# Editorial: Actuation, sensing and control systems for soft wearable assistive devices

**DOI:** 10.3389/frobt.2022.992699

**Published:** 2022-09-08

**Authors:** Jesús Ortiz, Giorgio Grioli, Jonathan Rossiter, Tim Helps, Ali Sadeghi, Michele Xiloyannis

**Affiliations:** ^1^ Italian Institute of Technology, Genoa, Italy; ^2^ Centro di Ricerca “E. Piaggio” of the University of Pisa, Pisa, Italy; ^3^ Department of Engineering Mathematics, University of Bristol, Bristol, United Kingdom; ^4^ Bristol Robotics Laboratory, University of Bristol, Bristol, United Kingdom; ^5^ Department of Biomechanical Engineering, University of Twente, Enschede, Netherlands; ^6^ SensoryMotor Systems (SMS) Lab, Institute of Robotics and Intelligent Systems (IRIS), ETH Zürich, Zürich, Switzerland; ^7^ Spinal Cord Injury Center, Balgrist University Hospital, Medical Faculty, University of Zurich, Zürich, Switzerland

**Keywords:** exoskeletons, exosuits, soft sensors, smart actuation, control

## 1 Introduction

In recent years the use of exoskeletons has garnered increased interest in various areas and sectors, from rehabilitation to industrial applications. Most previous developments have been based on classical robotics technologies and are typically built from rigid structures with electric or hydraulic actuation systems. While these are more mature technologies and these systems can offer a high degree of support, the drawbacks from the point of view of wearability and usability are difficult to overcome, mostly due to weight and comfort.

Recently, there have been several important developments in the area of soft robotics, including, but not limited to, sensing, actuation, and control, which enable the development of soft exoskeletons, also called exosuits. These devices are being developed for 1) military applications, for the improvement of the mobility capabilities of soldiers, 2) medical applications, for use assisting people with minor to moderate impairments, and 3) industrial applications, for the reduction of operator physical exertion.

However, it is still a very challenging task to develop an exosuit. On one side, the actuation system needs to be adapted to the soft characteristics of the exosuit. For example, classical actuation technologies can be combined with a cable-driven mechanism in order to transmit the assistive forces to the user. Pneumatic actuation is also commonly used, mostly because it is a more suitable technology by nature. Novel technologies based on smart materials (for example Shape Memory Alloys) are also possible; however, it is difficult to meet the assistance requirements with their performance. The second aspect is the sensing technologies which, due to the physical properties of the soft components, also present very important challenges. The particular characteristics of the sensors and actuators imply the need for a specific control system.

User interfaces that facilitate the use of wearable assistive devices must also be developed to ensure their success and adoption across industries. Additionally, very little is known about how such devices are used and perceived by users and organizations. Field and laboratory evaluations of these systems with human subjects using quantitative and/or qualitative methods can inform the future design of these technologies as well as help us understand their impact on human productivity and health and well-being.

## 2 Contributions

This Research Topic aimed to bring together the latest developments in soft exoskeletons to explore current trends in the different areas related to these technologies, in terms of actuation, sensing, and control. The issue includes five contributions, with a strong common point between them: all the papers target the assistance of the upper body: elbow (Missiroli et al.; Sambhav et al.), wrist (Chiaradia et al.) and hand/finger (Dickmann et al.; Dittli et al.). One possible reason for this trend is that generally speaking, the upper body and in particular wrist/hand/finger assistance requires less force/torque levels than lower body assistance, which needs to cope with full-body weight and inertia.

For the actuation systems, some of the contributions propose a cable-driven system (Missiroli et al.; Chiaradia et al.; Dittli et al.). Another article by Sambhav et al. proposes a cable-driven system, but this study is based on a simulation framework and not on a study with a physical prototype. Dickmann et al. proposes a more classical mechanical system. Other actuation systems using hydraulic, pneumatic, and other principles are not considered in the included papers. This is partially motivated by the fact that most of the proposed systems are fully working prototypes and include subject trials.

From the sensor point of view, the works included in this topic seem to agree on more mature technologies, namely IMUs and EMG. The focus of these studies relies not on the sensor technologies but on the processing of data, and the understanding of the models that govern human motion and intention (Missiroli et al.; Sambhav et al.).

The design and construction of the exosuits are also an important part of some of the contributions (Chiaradia et al.; Dickmann et al.; Dittli et al.), which highlights that the design of these devices is still an important challenge and important steps are being made toward more advanced and efficient structures.

## 3 Perspectives

The development of exosuits is a very challenging field, but research in this area is progressing very positively towards a better understanding of these systems. The papers included in this Research Topic are a very good example of this. The main technologies required for the development of exosuits have already reached a good level of maturity. This allows us to perform research not only on system development but also on system evaluation, and consequently improve our understanding of the interaction between a soft exoskeleton and the wearer, which in turn helps to further improve this technology.

The second main aspect highlighted in this Research Topic is that research on enabling technologies for exosuits is still very active. Traditional technologies on actuation, such as cable-driven systems, pneumatic, and sensing, such as IMUs and EMGs, are commonly used on exosuits. While these technologies allow a good level of development and integration, there is a wide number of alternative technologies that are not mature enough, but which could potentially lead to a higher level of integration with a soft device. Shape Memory Alloys, Electroactive Polymers, Electro/magnetorheological fluids/polymers technologies have been explored in recent years, but they still lack the minimum requirements for the development of a fully functional exosuit. Different technologies for the development of soft sensors that can be integrated into the garments have been also explored, but their performance and reliability are not yet at the level of more traditional and mature technologies.

Research on the area of exosuits in the next few years, in all its facets, will undoubtedly be very exciting, with a wider range of applications.

